# Simplified MR imaging of the inner ear in patients with Ménière's disease

**DOI:** 10.3389/fneur.2023.1289357

**Published:** 2023-10-05

**Authors:** Daphne J. Theodorou, Stavroula J. Theodorou, Ekaterini Ahnoula, Vasilios Mitsios

**Affiliations:** ^1^Department of Radiology, General Hospital of Ioannina, Ioannina, Greece; ^2^Department of Radiology, University Hospital of Ioannina, Ioannina, Greece; ^3^Department of Neurotology and Otolaryngology, Head and Neck Surgery Unit, General Hospital of Ioannina, Ioannina, Greece

**Keywords:** MRI, inner ear, labyrinth, Ménière disease, hearing loss

In a recent study of patients with Ménière disease (MD), Xia et al. (1) investigated the anatomical variations of the inner ear hypothesis relating to development of endolymphatic hydrops (ELH). These authors also examined the role abnormalities of the jugular bulb (JB) may play in the pathogenesis of ELH. The large series of patients and controls were thoroughly studied with computed tomography (CT) and radiological indices were compared between the MD ears and control ears. The authors concluded that the anatomical variations observed in the vestibular aqueduct (VA) rather than abnormalities in the JB may predispose patients to MD.

The VA is a bony canal in the osseous labyrinth containing the endolymphatic duct and sac. Enlargement of the VA has been deemed the most common congenital inner ear malformation that is also associated with sensorineural hearing loss (2, 3). CT is an imaging method with high spatial resolution that provides exceptional detail of bone, allowing for detailed depiction of the osseous VA at the expense of radiation. Furthermore, CT cannot show the membranous labyrinth including the VA. With MR imaging, the signal intensity for each tissue depends on the intrinsic composition of that tissue (i.e., fluid content) and on the radiofrequency pulse applied to the tissue to generate images. Because the membranous labyrinth (investing the osseous labyrinth) of the inner ear is filled with fluid (lymph), MR imaging has been typically used to delineate the vestibulocochlear lymph space (4). In this respect, previous researchers have used advanced gadolinium-enhanced MR imaging studies to investigate ELH, with emphasis on diagnosis of Ménière disease (5–7). Off-label use of high-dose gadolinium compounds that may be ototoxic, the intravenous or interventional, intratympanic administration of contrast material, delayed and time-consuming imaging that may be associated with enhancement of the surrounding structures (i.e., vessels), and high-strength 3.0 T MR units have arisen as certain limitations to routine clinical use of these imaging protocols, however (8).

In our clinical and imaging practices, we recommend that MR imaging be considered in patients with otovestibular symptoms, suggestive of MD. To this end, we have advocated a simple MR imaging protocol of the inner ear that would employ the 3D-constructive interference in steady state (3D-CISS) sequence (or equivalent fast gradient-echo pulse sequence). The CISS sequence is readily available in most high-strength (1.5 T or above) MR units and allows for fast acquisition of submillimeter high resolution images and multiplanar reconstructions that facilitate delineation of fine structures in the inner ear, and the JB. Because of a direct cisternographic effect that enables direct depiction of the vestibulocochlear lymph space, visualization of the minute anatomic structures in the membranous labyrinth (including the VA) and the acoustic nerves is feasible, sparing the administration of gadolinium material (9). Additional, post-processing maximum intensity projection (MIP) images with a slab thickness 4–5 mm generated form the original MR data may further increase conspicuity of the fine inner ear structures (Figure 1).

**Figure 1:**
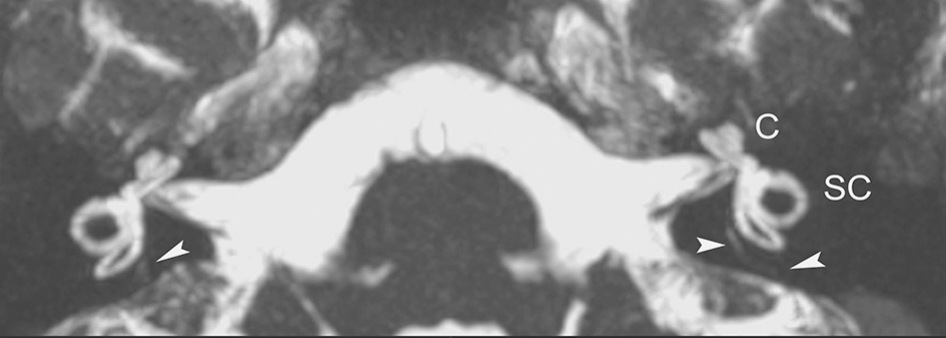
Axial non gadolinium-enhanced 3D-CISS image (with MIP technique) delineates normal anatomy of the inner auditory canal and the membranous labyrinthine structures on both sides, in a 16-year-old patient with vertigo and left ear tinnitus. At this image level, portions of the VA (arrowheads) can be seen. C, coclea; SC, semicircular canals.

We have found the single MR sequence approach both simple and efficacious and recommend this fast imaging survey for exploring inner ear abnormalities, in patients with symptoms that may be related to MD. Perhaps, the authors would agree with us that dedicated MR imaging protocols could be complementary to CT studies of the inner ear to acquire high resolution images of the integrated osseous and membranous labyrinthine anatomic structures. Besides, abnormalities of the osseous labyrinth may not be uncommon, especially in patients with otovestibular symptoms, and changes in the petrous bone structures (i.e., early labyrinthitis ossificans, otosclerosis) may need to be thoroughly studied on the high-resolution CT (HRCT) images.

## Author contributions

DT: Conceptualization, Formal analysis, Resources, Supervision, Validation, Writing—original draft, Writing—review and editing. ST: Conceptualization, Data curation, Formal analysis, Investigation, Methodology, Resources, Software, Validation, Writing—original draft, Writing—review and editing. VM: Data curation, Resources, Supervision, Validation, Visualization, Writing—review and editing.
